# Chitosan Hemostatic Dressings: Properties and Surgical Applications

**DOI:** 10.3390/polym16131770

**Published:** 2024-06-22

**Authors:** Roberta Cassano, Paolo Perri, Edoardo Scarcello, Paolo Piro, Roberta Sole, Federica Curcio, Sonia Trombino

**Affiliations:** 1 Department of Pharmacy, Health and Nutritional Science, University of Calabria, 87036 Rende, Italy; roberta.cassano@unical.it (R.C.); roberta.sole@unical.it (R.S.); 2 Department of Vascular and Endovascular Surgery, Annunziata Hospital, 1 Via Migliori, 87100 Cosenza, Italy; p.perri@aocs.it (P.P.); e.scarcello@aocs.it (E.S.); p.piro@aocs.it (P.P.)

**Keywords:** chitosan, hemostasis, hemostatic dressing, surgical application

## Abstract

Wounds caused by trauma and/or surgery represent a significant challenge in contemporary medical practice, requiring innovative approaches to promote optimal healing and reduce the risk of bleeding and complications resulting from it. In this context, chitosan, a natural polysaccharide derived from chitin, represents an ideal material for the study and application of medical devices, in the form of dressings, in wound management for pre- and/or post-operative wounds due to its ability to induce hemostasis and its high biocompatibility with biological tissues. The aim of this work was to discuss the structural characteristics, properties and application of chitosan-based hemostatic dressings in hemostatic processes resulting from pre- or post-surgical approaches.

## 1. Introduction

Bleeding due to injury, trauma, surgical procedures and coagulation disorders carries several health risks that require timely intervention [1,2]. In this regard, it is crucial to induce potentially life-saving effective hemostasis in apre-hospital setting, as well as in clinical and surgical treatment, as uncontrolled bleeding can cause various complications for both surgeons and patients [3]. For example, there can be adverseunfavorable intraoperative or perioperative outcomes, surgery and post-operative hospitalization lasting longer than expected, prolonged wound healing time, a high likelihood of developing infections and shock, the occurrence of hematoma and/or coagulopathy and increased morbidity and mortality. Time is therefore of the essence when treating serious injuries associated with massive hemorrhage, and saving time increases the chances of survival for patients in a hostile scenario or an emergency. To be effective in clinical practice, hemostatic dressings must work quickly [4]. Conventional techniques to achieve hemostasis in surgery include different mechanical approaches, using sutures, vascular clips, bone wax, standard dressings, etc., and thermal approaches, such as electrocautery [5]. In order to activate hemostasis processes, especially in asurgical environment, topical hemostatic products such as gauze and plasters are used in combination with these methods. They are able to control blood loss and minimize the risk of associated complications and subsequent mortality and morbidity [6]. However, these conventionally defined topical hemostatic agents provide low skin irritation and good breathability but adhere poorly to wounds, have a slow hemostatic capacity and absorb too much blood, which often causes further bleeding [7]. For this reason, a functional hemostatic dressing should possess properties such as the ability to stop bleeding within two minutes, adequate biodegradability and bioabsorbability, clinical safety, ease of use and low cost [8]. Nowadays, there is a wide range of new-generation topical hemostatic agents available with features that improve their performance in the clinic and surgical fields [9]. They possess greater flexibility and firm adhesion to the surface of injured tissues; a rapid ability to induce hemostasis, even in patients taking anticoagulants or found to be coagulopathic; they are painless and they leave no residue [10]. In this review, we focus on topical hemostatic agents based on chitosan, a naturally occurring polymer with excellent hemostatic, antibacterial and antifungal properties and a high degree of biocompatibility, biodegradability, non-immunogenicity and healing effects [11,12]. It is important to underline that most biological and chemical applications of chitosan, especially in terms of hemostasis, are based on its cationic properties and its versatility as a biomaterial [13,14]. Indeed, by coming into contact with negatively charged platelet and erythrocyte surfaces, chitosan is able to stop bleeding by absorbing water and converting it into an adhesive element that adheres to the damaged tissue [15]. The chitosan-based hemostatic dressings currently commercially available, and widely used in aclinical setting, are ChitoGauze, CeloxGauze, HemCon, Trauma Gauze and ChitoFlex. These differ both in the mechanisms of action they exert to activate coagulation (adsorption of fluid, cross-linking of red blood cells, formation of a mucoadhesive barrier) and in the form given to the hemostatic agent (granular rolled gauze, flexible rolled bandage, etc.), which may influence the usefulness of the hemostatic agent fordifferent types of wounds [16,17]. The aim of the present work was to discuss the structural characteristics, properties and application of new-generation chitosan-based hemostatic patches, currently commercially available, to induce hemostasis in surgical and non-surgical treatments.

## 2. Chitosan-Activated Hemostasis

Chitosan is a copolymer of N-acetyl-D-glucosamine and D-glucosamine containing a varying number of N-acetyl groups obtained through the deacetylation of chitin, gene-rally extracted from the exoskeleton of crustaceans, with basic aqueous solution using sodium hydroxide [18]. It is a substance insoluble in water and/or organic solvents but soluble in acidic solutions. When considering the application of chitosan in the biomedical field, it is necessary to take into account the source of its derivation and the extraction process of the polymer, which determine the characteristics of the end product. Properties such as purity, crystallinity, molecular weight (Mw) and degree of deacetylation (DD) are particularly significant, as they are related to the mechanical and biological properties of chitosan [19,20]. In addition, chitosan having different molecular weights (Mws) and different degrees of deacetylation (DDAs) gives the material hemostatic capacity [21]. A high degree of deacetylation/DDA promotes the aggregation of erythrocytes and platelets, which is necessary to initiate hemostasis [22]. The acquisition of a positive charge by chitosan occurs when N-acetyl D-glucosamine is deacetylated to form D-glucosamine, providing free amine groups on its molecular structure. It is the cationic form of chitosan that is exploited to aid hemostasis, as platelets and erythrocytes are negatively charged due to the presence of phosphatidylcholine, phosphatidylethanolamine and sialic acid groups. The amine groups present in chitosan play a crucial role in facilitating erythrocyte aggregation through electrostatic interactions with cell surface charges. Specifically, the hemostatic effect of chitosan is achieved through the overall action of these mechanisms [23]:

(i). The presence of opposite charges between chitosan and erythrocytes causes positively charged glucosamine to attract negatively charged red blood cells, causing agglutination and promoting coagulation [24]. In particular, the binding that chitosan establishes with red blood cells may be related to the increase in its molecular weight and the degree of entanglement due to the particular intermolecular hydrogen bonding force or the electrostatic repulsion between the polyelectrolyte molecules [25].
(ii). Stimulating platelet adhesion and aggregation is not straight forward, as it depends on a number of properties, including mobility of the surface chains, surface chemical composition, hydrogen bonding properties, charge density and hydrophobicity/hydrophilicity [26].
(iii). By exploiting covalent/hydrogen bonds and reversible hydrophobic interactions between chitosan and plasma proteins (interpolymer complexation), it is possible to obtain an effective physical barrier, defined as a blood protein–membrane barrier, directly at the bleeding site. This process goes beyond normal blood coagulation, as it activates independent coagulation, which is particularly suitable for patients with coagulopathy [27,28].
(iv). There is an alteration of the fibrinogen structure associated with electrostatic forces after ionization of the polymer itself [29,30].

## 3. Chitosan Hemostatic Dressings

The chitosan-based plasters considered in this review act as hemostatic agents by exploiting the chemical–physical characteristics of the polymer with which they are made [31]; see Table 1. For example, HemCon^®^ patches (HemConMedical Technologies, Inc., Portland, OR, USA), of which there are several variants, consist of lyophilized chitosan acetate salt that exerts its hemostatic action by improving platelet function; Celox™ patches (MedTrade Products Ltd., Crewe, UK) consist of chitosan granules or flakes that interact with red blood cells to form a strong plug; Axiostat patches consist of a porous sponge of chitosan that acts through a mechanism of mucus adhesion to the injured tissue; TraumaStat patches consist of freeze-dried chitosan, containing highly porous silica and polyethylene, ideal for the control of moderate to severe bleeding. Chitosan patches are also chitosan-based but have a cellulose backing that reduces the compression time when used to stop bleeding [32]. It is important to specify that when a dressing composed of chitosan is applied to a bleeding wound subjected to ‘opposing pressure’, which can be applied manually or by packing, the blood flow through the gauze is slowed down and becomes denser due to the highwater absorption of the gauze. Meanwhile, the red blood cells adhere to the chitosan threads due to the Coulomb attractive force because they have opposite charges, coating one or, at most, several layers of red blood cells on the gauze. At the same time, H+ ions are continuously released from the chitosan (associated with NH_3_ molecules) into the blood, reducing the negative charges on the surrounding red blood cells (associated with COO molecules) and thus the repulsive electrical force of the double layer between the red blood cells [33]. As a result, the red blood cells in the vicinity of the gauze aggregate with each other and adhere to the layer(s) previously smeared on the gauze. The aggregation process continues as more H+ ions are released, forming a layer-by-layer structure by shrinking the grid space in the gauze over time, and eventually, a mucoadhesive barrier’ is formed, and bleeding is stopped [34]. The technology of chitosan-based dressings, examined in this review, lies mainly in maximizing the bioadhesive properties of native chitosan without chemical modifications in its backbone. Indeed, it is well known that chitosan obtains its cationic charge through the protonation of the primary amine groups (NH_2_) present in its glucosamine subunits. However, this protonation is pH-dependent; at a pH above 6.5, chitosan undergoes neutralization, while at a physiological pH of 7.4, it loses its positive charge completely. The hemostatic activity of such dressings relies solely on the physical properties and microscopic morphology of this natural material to maximize its cationic charge.Its uniform microscopic porous structure provides a unique molecular chemistry of the chitosan within the matrix, which helps to maintain its positive charge for a prolonged duration and even under physiological conditions. For example, Axiostat’s highly porous structure helps to improve tissue–biomaterial interaction at both the macro and micro scale. In fact, when Axiostat is pressed against injured tissue, it creates a gap within its pores that leads to mechanical interlocking with the tissue surface, providing instant bioadhesion. In addition, the high porosity of Axiostat promotes the diffusion of surface-bound anionic molecules into the cationic pores of the dressing itself, which results in diffusive adhesion, a mechanism in which surface-bound molecules from tissues diffuse into the pores of the dressing to form strong bioadhesion. Through this mechanism, such dressings are able to maintain their positive charge and provide strong mucoadhesion even under physiological conditions with blood components. Indeed, red blood cells (RBCs) and platelets are trapped in the porous structure, which leads to platelet activation and the formation of a massive blood clot [35,36].

### 3.1. HemCon Patches

**Figure 1:**
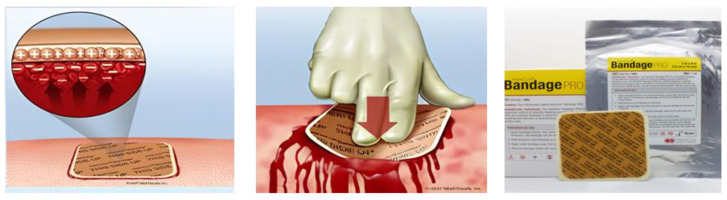
Schematic representation of the application and the hemostatic process activated by HemCon dressing.

HemCon dressings were the first patches to be approved by the FDA as hemostatic devices, useful especially in war zones. In fact, the marketing of this device in 2003 was initially mainly for US soldiers on the battlefields of Afghanistan and Iraq [37]. Indeed, there was a need in combat zones to provide a hemostatic product with direct pressure that could quickly control and stabilize hemorrhages that could not be treated with a simple tourniquet to ensure the transportation of wounded soldiers. The benefits obtained made it possible to extend the use of this patch to civilian use as well (Figure 1). However, the original formulation of the HemCon plaster was not suitable for the treatment of irregular and deep wounds due to its rigidity and for the treatment of smaller wounds without the danger of cutting or tearing [38]. In this regard, research has improved its pliability by adding a chitosan-based gauze roll that allowsit to be packed into wounds to ensure hemostasis through stimulation and platelet adhesion. Hence, new forms of HemCon^®^ based on chitosanhave been developed, includingHemCon Patch^®^ Pro, HemCon^®^ Bandage, HemConChitoFlex^®^, HemCon^®^ GuardaCare PRO, etc.

#### 3.1.1. HemCon Patch^®^ Pro and HemCon^®^ Bandage

This is a hemostatic dressing useful mainly for external and temporary control of severe bleeding wounds that also require antibacterial action. In contact with anionic erythrocytes, the chitosan salts of the device ‘cross-link’ rapidly, adhering strongly to the wound surface [39]. HemCon Patch^®^ Pro also promotes the formation of an antibacterial barrier against *Staphylococcus aureus* (MRSA), *Enterococcus faecalis* (VRE) and *Acinetobacter baumannii*, thanks to the action of chitosan, which destroys the bacteria’s cell membranes [40]. Chitosan can bind to DNA, penetrate bacterial cells and inhibit mRNA synthesis and DNA transcription or bind directly with the negatively charged components of bacterial cell walls, forming a resistant layer around the cell and hindering ion transport into the bacterial cell itself, causing its death [41,42]. It also has a flexible, contoured design that maintains structural integrity, as it does not break, crumble or peel away from the wound, creating a stable clot and providing localized support for coagulation [43]. It is widely used in post-catheterization procedures and electrophysiology laboratory procedures and controls bleeding in hemodialysis patients; it is also indicated to control skin bleeding during percutaneous needle access. The application of this dressing requires simple steps that ensure its high safety and efficacy. Before use, the following procedure is necessary:

- Remove the sheath following hospital protocol.
- Allow a small amount of blood to leak out to surround the puncture site.
- Do not clean the puncture site or moisten it with saline solution. Blood isneeded to facilitate the adhesionprocess.
- With the printed side facing up, place HemCon Patch^®^ PRO directly over the puncture site. The patch can be cut to size. Do not remove the backing.
- Maintain digital pressure along the entire vascular access tract until bleeding is controlled.
- The patch will adhere to the site where bleeding is present [44].

#### 3.1.2. HemCon ChitoFlex

HemCon ChitoFlex is a chitosan dressing made up of several layers of polymer rolled into a device that can be inserted into a wound in the same manner as regular gauze. The hemostatic mechanism exerted is based on the action exerted by chitosan on red blood cells and platelets, which, through cross-linking, leads to the formation of a mucoadhesive physical barrier around the site of bleeding [45]. Unlike HemCon, ChitoFlex is composed of chitosan on both sides of the product, so it is necessary to wrap it around itself several times in order to treat hard-to-reach wounds and it therefore cannot provide a continuous interface with the injured vessels. This dressing works well in pathological conditions where there is low pressure and no blood supply, whereas in situations where pressure is high, this device tends to break down over time and promote new bleeding. The increase in blood pressure during the resuscitation phase could act on the mucoadhesion mechanism of the dressing [46].

### 3.2. Celox

This is a dressing consisting of chitosan granules and flakes, which provide a high surface area of contact with blood. In general, chitosan, in contact with aqueous systems and physiological environments, tends to absorb large amounts of the aqueous phase, which leads to its structural modification. This phenomenon underlies the hemostatic mechanism that this dressing activates when it is placed on a bleeding site [47]. In detail, the interaction between blood and Celox causes the swelling of the granules by hydrogel absorption, resulting in the formation of a sticky granular gelatinous substance that is difficult to completely remove given the wide spread of granules in the wound [48]. Furthermore, because the hemostatic mechanism of chitosan is independent of the physiologic mechanisms that regulate coagulation, this dressing has been shown to be highly effective in coagulopathic patients undergoing therapy with common antithrombotic drugs such as heparin or warfarin [49]. As it does not generate heat, unlike many mineral-derived hemostatic agents, this device is equally useful in inducing hemostasis in individuals with severe hypothermia (a body temperature below 29° centigrade), in whom blood struggles to clot, often as a consequence of hypovolemic shock [50]. Celox is widely used to promote hemostasis in heavily bleeding wounds such as bruised liver injury, arterial puncture bleeding, groin laceration, etc.

### 3.3. Axiostat

**Figure 2:**
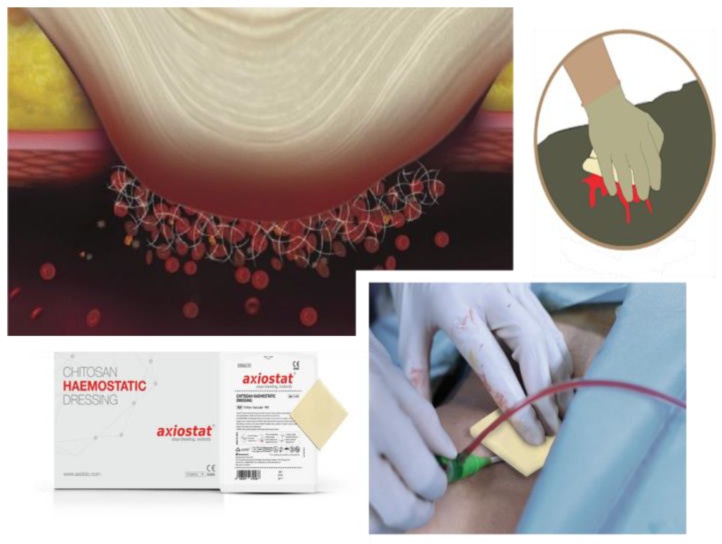
Axiostat dressing use and application.

This is a sterile, single-use, nonabsorbable dressing consisting of a highly porous chitosan matrix with a honeycomb microstructure [51]. This conformation allows for the rapid absorption of blood components within the dressing, resulting in the accumulation of erythrocytes and platelets at the wound site and the formation of a strong blood clot in 2 to 3 min [52]. This mechanism, termed mucus adhesion, promotes the formation of a mechanical barrier over the bleeding site that leads to fibrin formation, creating a plug that extends throughout the wound site, thus stopping the bleeding. The wound remains closed thanks to the fibrin network (Figure 2) [53]. This dressing is primarily indicated for vascular procedure sites; sites involving percutaneous catheters, tubes and pins; lacerations and abrasions; skin surface puncture sites; surgical debridement sites; to control oral bleeding during extraction or other dental procedures and for trauma and maxillofacial surgery [54]. The use of Axiostat involves removing excess blood around the wound; placing gauze that is approximately the size of Axiostat patch itself; shaping the dressing to the size of the wound and placing Axiostat on the wound using the three-finger technique. Gently easing the pressure applied allows a small amount of blood to flow from the affected site, precisely because the Axiostat patch’s mechanism of action is activated when it comes into contact with blood [55]. Pressure must then be re-established over the bleeding site by holding the patch firmly, but without applying excessive pressure, until the bleeding stops. If bleeding is not controlled or the dressing is completely saturated with blood, another device must be used. In addition, a secondary dressing should be applied to ensure that it remains in place and to prevent wound contamination [56]. After the hemostatic effect has been explored, it is necessary to remove the dressing through irrigation with water or saline solution; in this way, it turns into a liquid gel that can be removed easily, and the removal is painless without causing further bleeding [57].

### 3.4. TraumaStat

TraumaStat^®^ is a hemostatic gauze consisting of chitosan, silicon dioxide (which acts as a robust activator of the intrinsic coagulation cascade) and polyethylene [58]. In detail, this dressing is composed of permeable polyethylene fibers, 20 μm in diameter, coated with chitosan and filled with precipitated silica [59]. It has a larger specific surface area, which significantly increases the wound contact area and enables strong interaction with platelets and coagulation factors by increasing the rate and strength of the resulting clot. The hemostatic mechanism exerted relies on the mobilization of Ca^2+^ to increase platelet activation and coagulation factor activation for clot formation. TraumaStat^®^ is designed for temporary external use, aiming to effectively control moderate to severe bleeding [60].

### 3.5. ChitoSeal

This is a dressing made up of chitosan and cellulose, useful in reducing the time needed for compression. it is especially effective in managing bleeding at vascular access sites and around catheters or percutaneous tubes. ChitoSeal was introduced in catheterization laboratories to reduce the occurrence of hematomas, to ensure faster and/or earlier ambulation times and to reduce the time needed for hemostasis [61]. Specifically, ambulation occurs after 3 to 4 h, instead of after 6 h with manual compression.

## 4. Surgical Applications of Chitosan Dressings

Surgical interventions require maintaining a clean view of the operating field and the hemodynamic balance of the patient and reducing procedure times, anesthesia and complications [62,63]. Excessive bleeding, in fact, can be responsible for large blood losses, which require timely transfusions of blood or blood products, exposing the patient to immunological, infectious and immunosuppressive complications [64,65]. Preventing excessive blood loss during surgery is equivalent to significantly reducing the risk of perioperative complications and surgical reoperation, thus reducing hospital stays and hospitalization costs [66,67]. In a study conducted by Pappas et al. on venous and/or arterial femoral sheaths undergoing elective percutaneous diagnostic or therapeutic procedures, it was shown that the use of the HemCon patch successfully reduced patients’ waiting time and hospital stay by promoting patients’ ambulation four hours after surgery without complications [68]. The flexibility of such a patch proved advantageous because it conformed perfectly to the site of the lesion, allowing for greater control at the puncture site and reducing the risk of bleeding. A key difference observed by the research team was the ability to feel the arterial pulse through the patch. HemCon bandage patches act as a backup to traditional closure devices because they reduce the localized pain associated with the presence of a foreign body (the closure device) in the groin area and exert antibacterial action. In another study of 60 patients undergoing hemodialysis via arteriovenous fistula, the application of a HemCon dressing versus conventional gauze was evaluated on a bleeding wound resulting from an access puncture. The results obtained showed that in 76.6% of cases, hemostasis was achieved at the arterial access site within the first two minutes, while in patients treated with the conventional dressing, the average time to homeostasis was 10 to 12 min. The flexibility of such a patch proved advantageous, as it conformed well to the bleeding site [69]. Binnetoglu et al. conducted a study to compare the efficacy of the hemostatic agent Algan (AHA) with Celox in 28 albino Wistar rats after experimental femoral artery injury. The results obtained showed that compared with the control group, AHA and Celox were very effective in controlling bleeding (*p* < 0.001) by providing effective hemostasis within 2 min after application [70]. Chitosan dressings are widely used in surgical settings to facilitate closure with manual compression of the femoral arterial access site in patients undergoing endovascular treatment. In these cases, after the removal of arterial introducer sheaths, it is necessary to medicate the vascular access site by manual compression (MC) or using a vascular closure device (VCD) to promote hemostasis and avoid serious complications (hematomas, recurrent bleeding, pseudoaneurysms, vessel thrombosis, infection and wound dehiscence). In this regard, a study conducted by Minici et al. involved 120 patients undergoing manual compression closure of the femoral arterial access site with the help of the Axiostat hemostatic dressing after femoral artery surgery at a specific time interval. The results showed that primary technical success was achieved in 91.7% of patients, with adequate hemostasis achieved after around 8.9 (±3.9) min and ambulation occurring after 462 (±199) min. Bleeding-related complications were observed in only 5.8% of them [71]. Axiostat is also safe and effective in dental surgery, especially in coagulopathic patients in whom early reflex vasoconstriction occurs because of the narrowing of the vascular smooth muscle cells, but being a transient process, bleeding will persist if fibrin is not formed [72]. The chitosan in the Axiostat dressing induces platelet activation and the formation of a stable clot that seals the area and significantly reduces the bleeding time [73]. In a prospective study conducted by Rajendra et al., the efficacy of controlling hemostasis in cardiac patients undergoing antiplatelet therapy and dental extraction was evaluated. From the results obtained, Axiostat was found to be of effective value in reducing the bleeding time in patients treated after dental surgery. In addition, a reduction in post-operative pain and comorbid conditions was observed [74].

## 5. Conclusions and Future Perspectives

The use of chitosan-based systems to control hemostasis has enabled the development and commercialization of several formulations and devices used in surgery. Specifically, in this work, chitosan-based dressings were analyzed and described as highly biocompatible devices, with hemostasis, fluid absorption and inhibition of microbial proliferation. Since the procoagulant characteristics of chitosan are fundamental to obtaining hemostatic effects, the advantages deriving from dressings based on this biopolymer compared to conventional gauzes have been analyzed in the literature by several authors. This review demonstrated not only that the use of this dressing in the management and control of hemostasis in post-operative and major trauma surgical hemorrhage was effective but also that the mean time to hemostasis was significantly reduced with chitosan drugs, as well as the incidence of adverse events (hematomas, pseudo aneurysms, infections, etc.) being significantly lower. In addition, the reduced hospitalization time and the speed of patient recovery make chitosan dressings innovative devices to quickly stop bleeding in sites of hemorrhagic lesions without the use of stitches or conventional dressings. However, to date, there is no specific hemostatic product on the market that can be considered ideal, but nanotechnology is continuously being developed to improve and design hemostatic materials with increasingly effective and biosensitive optimal properties that can rapidly stop bleeding. In this regard, by mixing chitosan with functional substances exhibiting pharmacological and/or biological activity (painkillers, anti-inflammatories and healing materials), it is possible to obtain composite materials capable of providing increasingly rapid hemostasis through the activation of synergistic and non-synergistic mechanisms, such as those already explained for this biopolymer.

## Figures and Tables

**Table 1:** Chitosan hemostatic dressing classification.

Commercial Name	Form	Composition	Mode of Action
HemCon	Rigid and hard sponge bandage	Non-woven chitosan fibers	Amine groups on chitosan interact with RBCs and induce clotting
HemCon Patch^®^ Pro	Flexible and contouring dressing	Chitosan acetate	With anionic erythrocytes, the chitosan salts of the device ‘cross-link’ rapidly, adhering strongly to the wound surface
HemCon^®^ Bandage	Impregnated dressing	Chitosan acetate	Freeze-dried chitosan acetate salt, mainly used for emergencies to stop blood loss, enhance platelet function
HemCon ChitoFlex	Rolled chitosan gauze	Chitosan	Chitosan binds to red blood cells and platelets and forms a mucoadhesive physical barrier around the bleeding site
Celox	Dressing based on chitosan granules and flake form	Chitosan	Swelling of the chitosan granules
Axiostat	Highly porous chitosan matrix	Chitosan	Electrostatic interaction between positively charged biopolymer of Axiostat and the negatively charged blood cells
TraumaStat	Chitosan gauze	Polyethylene fibers, coated with chitosan and filled with precipitated silica	Mobilization of Ca^2+^ to increase platelet activation and coagulation factor activation for clot formation
ChitoSeal	Chitosan dressing	Supported with a cellulose coating	Amine groups of chitosan interact with RBCs and induce clotting

## Data Availability

The data are contained within the article.
